# Coffee as a dietary strategy to prevent SARS-CoV-2 infection

**DOI:** 10.1186/s13578-023-01154-9

**Published:** 2023-11-14

**Authors:** Chen-Shiou Wu, Yi-Chuan Li, Shin-Lei Peng, Chung-Yu Chen, Hsiao-Fan Chen, Po-Ren Hsueh, Wei-Jan Wang, Yen-Yi Liu, Ciao-Ling Jiang, Wei-Chao Chang, Shao-Chun Wang, Mien-Chie Hung

**Affiliations:** 1 Present Address: Graduate Institute of Biomedical Sciences, China Medical University, Taichung, Taiwan; 2 Research Center for Cancer Biology, China Medical University, Taichung, Taiwan; 3 Department of Biological Science and Technology, China Medical University, Taichung, Taiwan; 4 Department of Biomedical Imaging and Radiological Science, China Medical University, Taichung, Taiwan; 5 Neuroscience and Brain Disease Center, China Medical University, Taichung, Taiwan; 6 Departments of Laboratory Medicine and Internal Medicine, School of Medicine, China Medical University Hospital, China Medical University Taichung, Taichung, Taiwan; 7 Department of Biology, National Changhua University of Education, Changhua, Taiwan; 8 Center for Molecular Medicine, China Medical University Hospital, China Medical University, Taichung, Taiwan; 9 Department of Biotechnology, Asia University, Taichung, Taiwan; 10 Cancer Biology and Precision Therapeutics Center, China Medical University, Taichung, Taiwan; 11 Institute of Biochemistry and Molecular Biology, China Medical University, Taichung, Taiwan

**Keywords:** Coffee, SARS-CoV-2, ACE2, TMPRSS2, Cathepsin L

## Abstract

**Background:**

To date, most countries lifted the restriction requirement and coexisted with SARS-CoV-2. Thus, dietary behavior for preventing SARS-CoV-2 infection becomes an interesting issue on a daily basis. Coffee consumption is connected with reduced COVID-19 risk and correlated to COVID-19 severity. However, the mechanisms of coffee for the reduction of COVID-19 risk are still unclear.

**Results:**

Here, we identified that coffee can inhibit multiple variants of the SARS-CoV-2 infection by restraining the binding of the SARS-CoV-2 spike protein to human angiotensin-converting enzyme 2 (ACE2), and reducing transmembrane serine protease 2 (TMPRSS2) and cathepsin L (CTSL) activity. Then, we used the method of "Here" (HRMS-exploring-recombination-examining) and found that isochlorogenic acid A, B, and C of coffee ingredients showed their potential to inhibit SARS-CoV-2 infection (inhibitory efficiency 43–54%). In addition, decaffeinated coffee still preserves inhibitory activity against SARS-CoV-2. Finally, in a human trial of 64 subjects, we identified that coffee consumption (approximately 1–2 cups/day) is sufficient to inhibit infection of multiple variants of SARS-CoV-2 entry, suggesting coffee could be a dietary strategy to prevent SARS-CoV2 infection.

**Conclusions:**

This study verified moderate coffee consumption, including decaffeination, can provide a new guideline for the prevention of SARS-CoV-2. Based on the results, we also suggest a coffee-drinking plan for people to prevent infection in the post-COVID-19 era.

**Supplementary Information:**

The online version contains supplementary material available at 10.1186/s13578-023-01154-9.

## Introduction

Between the end of 2020 and nowadays, variants of severe acute respiratory syndrome coronavirus 2 (SARS-CoV-2) have been extending to different parts of the world [1]. Several reliable reports declared that there are at least 32 mutations in the spike protein of the Omicron variant twice that of the Delta variant [2–4]. Omicron has a complex series of genetic mutations that affect virus binding and immune evasion, such as enhancing ACE2 binding through RBD mutations [5], and achieving immune evasion through the combination of shared RBD mutations with specific mutations or NTD mutations [6, 7]. Furthermore, the optimization of the Omicron spike may make easier cellular entry through endocytosis, and the entry route of the Omicron has changed from relying on surface fusion via TMPRSS2 to endocytic fusion [8, 9]. These factors contribute to Omicron exhibiting higher properties in terms of transmissibility and immune evasion. Over time, the protective efficacy of current COVID-19 vaccines has faced frightful challenges, with countries around the world implementing boosters to combat the threat of rising COVID-19 cases [10, 11]. In addition to the latest preventive operations and strategies for treating COVID-19, diet is an environmental element that can impact the efficiency of SARS-CoV-2 infection. Recent research proposed that regular physical activity and a dietary intake with plentiful polyphenols [12–14] might induce active immune intervention, ameliorate immune escape properties to combat SARS-CoV-2 infection, and lower the risk of severe COVID-19 [15–17]. Probably, it's one of the best public health strategies to restrict the looming COVID-19 pandemic. One of the extreme widely consumed polyphenol beverages around the world is coffee. Coffee is a multiplex mixture of polyphenolic compounds, mainly including chlorogenic acid (CGA), caffeic acid (CAA), and additional antioxidant compounds such as cafestol, melanoidins, and trigonelline [18–20]. In previous literature, coffee is the dominant source of CGA, which can interfere with human blood pressure, lipid profile, glycemia and insulin resistance, to effectively ameliorate metabolic syndrome [21]. CGA also refined metabolism, inflammation, cardiovascular, and liver function in rats by enhancing the diversity of gut microbiota [22]. The overall efficacy of coffee has also been shown to exhibit antioxidant or repair mechanisms, anti-inflammatory, and anti-cancer capacity [23–25]. Vu et al. studied population data on the part of specific dietary intake in preventing COVID-19, and analyzed data of participants (n = 37,988) from the UK Biobank (UKB) to associate specific dietary behaviors with COVID-19. They found that drinking one or more cups of coffee per day was related to an approximately 10% lower risk of COVID-19 compared to less than 1 cup of coffee [26]. In human trial data of elderly volunteers aged 75–90 living in Madrid, Spain, the severity of COVID-19 was found to be significantly inversely associated with coffee consumption [27]. However, the protective mechanism of coffee against COVID-19 is unclear and worth studying. Therefore, we verified whether coffee has efficacy against SARS-CoV-2 through in vitro cell experiments, molecular docking, and the human trial study. During the spreading period of the Omicron variant, most of the positive cases had mild symptoms, and without taking anti-SARS-CoV-2 drugs [28, 29]. Regular dietary behavior to prevent SARS-CoV-2 infection becomes an interesting issue. In particular, coffee is one of the most common beverages people consume. The results from the current study may provide the scientific basis for the public to prevent from infection of SARS-CoV-2 by drinking coffee.

## Results

### Coffee is able to suppress the infection with SARS-CoV-2 variants

**Fig. 1:**
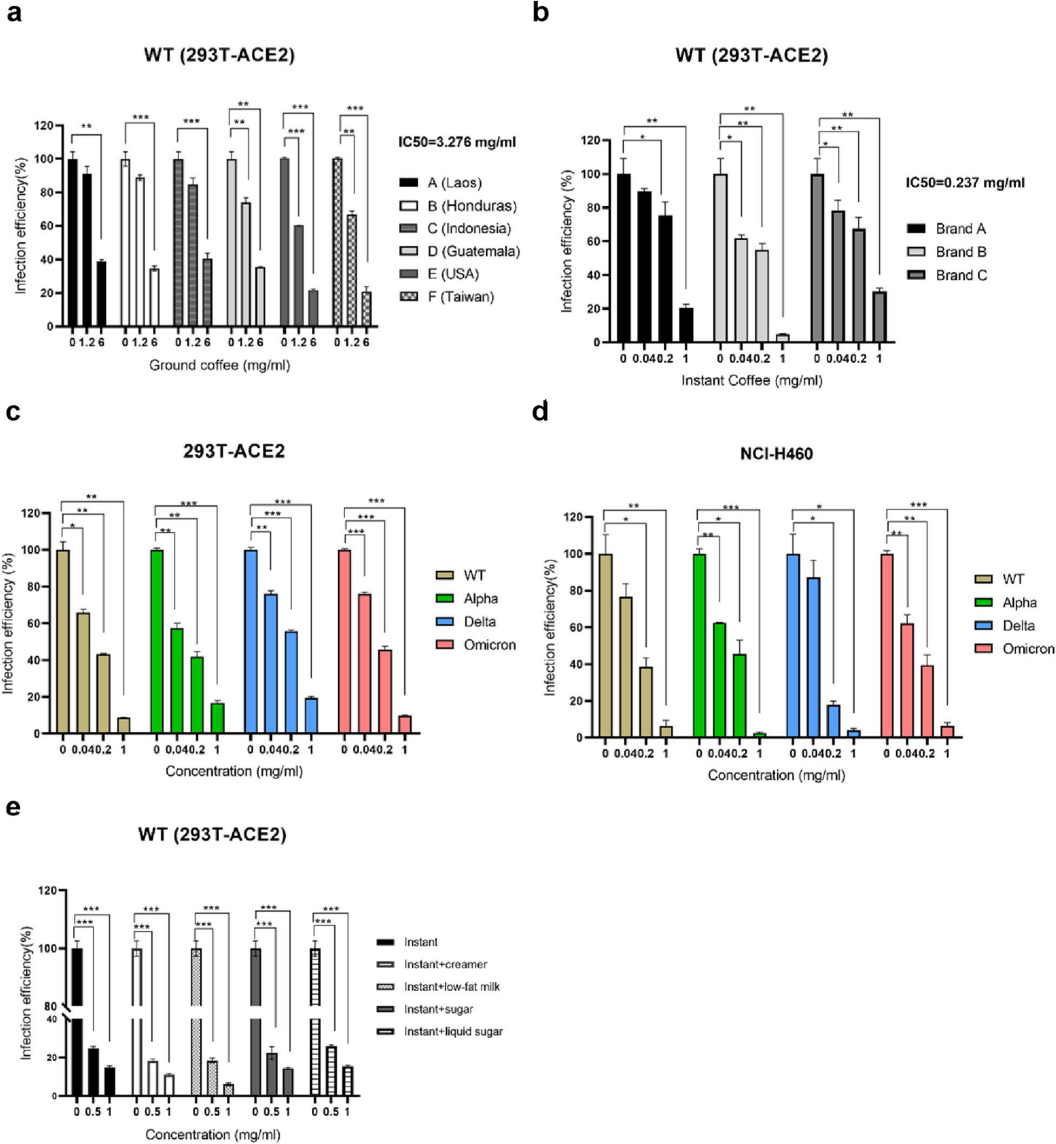
Coffee has inhibition on SARS-CoV-2 infection. **a** The inhibiting effectiveness of various races of ground coffee in different concentrations (weight of coffee grinding powder/ water volume) was tested on 293 T-ACE2 cells by using the wild-type (WT) SARS-CoV-2 viral pseudoparticle (Vpp) infection assay. **b** The percentage of inhibiting infection of different brands of instant coffee in different concentrations (coffee extract/volume) was examined by wild-type SARS-CoV-2 Vpp. **c** Testing the capability of instant coffee against wild-type, Alpha, Delta, and Omicron variants on 293 T-ACE2 cells by SARS-CoV-2 Vpp. **d** Examining the percentage of inhibition ability of instant coffee against wild-type, Alpha, Delta, and Omicron variants on NCI-H460 cells by SARS-CoV-2 Vpp. **e** The inhibiting SARS-CoV-2 entry ability of the instant coffee-added ingredient was checked by wild-type SARS-CoV-2 Vpp on 293 T-ACE2 cells. SARS-CoV-2 viral pseudoparticle (Vpp) infection assay (MOI = 0.1). Student T-test was performed for statistical analyses. Error bars indicated the standard error of the mean in triplicate independent experiments. Statistical significance was concerned as *P < 0.05, **P < 0.01 or ***P < 0.001

Based on the previous report analysis [26], drinking one or more cups of coffee per day was related to approximately 10% lower risk of COVID-19 compared to no coffee in the UK Biobank (UKB), we set to understand the impact of coffee on SARS-CoV-2 infection. With regards to this, we collected commercially available coffee beans produced from different places and measured their effects on the entry of SARS-CoV-2 by Vpp assay in the 293T-ACE2 cell line (human embryonic kidney cells transfected with ACE2 expression vector for expressing ACE2), which are sensitive to test infection efficiency through spike and ACE2 interaction. We observed that ground coffee at 6 mg/ml (weight of coffee grinding powder/ water volume) has the effect of reducing the entry of SARS-CoV-2 into host cells with an inhibitory of about 60 to 81% and presented a dose-dependent manner (Fig. 1a). Besides commercially available coffee beans, we further extended to verify whether commercially available instant coffee also had the potency to suppress the prospect of infection with SARS-CoV-2. The result showed that different brands of instant coffee significantly inhibit cell entry of SARS-CoV-2 at 1 mg/ml (coffee extract powder/ water volume), its inhibition efficacy (70–96%), and half maximal inhibitory concentration (IC50 = 0.237 mg/ml) (Fig. 1b). The cell viability was shown in Additional file 1: Figure S1 to validate that these concentrations of coffee have no killing effect on the cultured cells. Next, we used instant coffee to test whether it could also inhibit the entry of other SARS-CoV-2 variants into host cells. The data of the Vpp test validated that coffee at 1 mg/ml restricts the infection (inhibition efficacy about 81 to 97%) of the Alpha, Delta, and Omicron variants from entering 293T-ACE2 and NCI-H460 cells (Non-small-cell lung cancer) (Fig. 1c and d). With an additional question, whether adding milk or sugar to coffee affect its ability to combat SARS-CoV-2? It is well known that milk might reduce SARS-CoV-2 infection [30]. Therefore, we evaluated instant coffee including different additives by Vpp and found that instant coffee presented dose-dependent suppression of the SARS-CoV-2 entry, and adding different additives to coffee does not enhance its ability to inhibit the entry of SARS-CoV-2 into host cells (Fig. 1e). Together, these results indicated both ground and instant coffee have activities to reduce SARS-CoV-2 variants infection, and the suppressive activity was not affected by coffee additives.

### Coffee blocks SARS-CoV-2 infection through suppressing the binding of the SARS-CoV-2 spike protein to the host ACE2 and decreasing the activity of host TMPRSS2 and cathepsin L

**Fig. 2:**
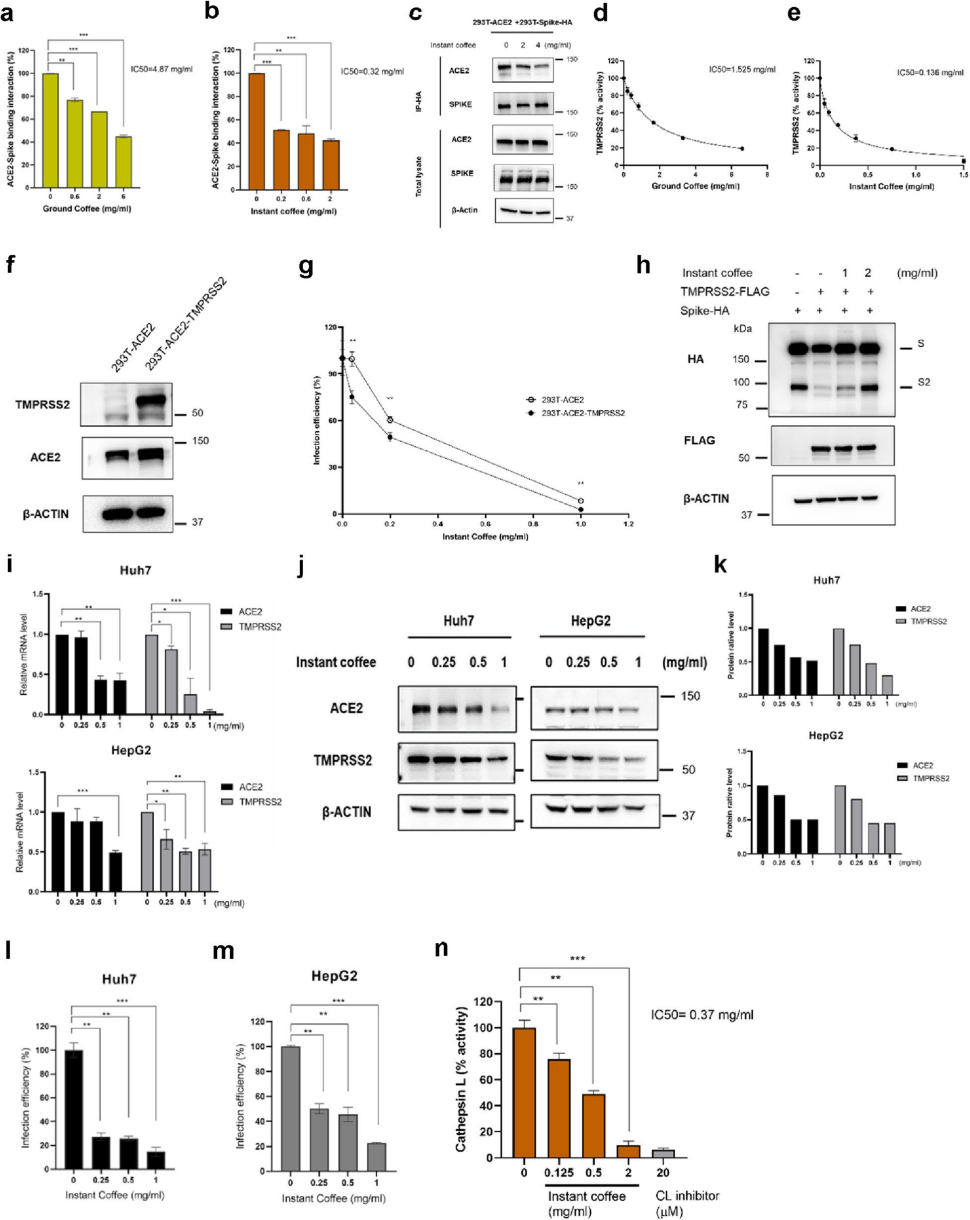
The mechanism of coffee blocking SARS-CoV-2 entry was identified. **a** The activity of ground coffee and **b** instant coffee in different concentrations to block SARS-CoV-2 spike-ACE2 interaction was measured by ELISA. **c** Co-immunoprecipitation using 293 T cells expressing SARS-CoV-2 spike-HA and ACE2 to test coffee can inhibit the binding of ACE2 and spike. After anti-HA pulldown, adding instant coffee (2, or 4 mg/ml), then ACE2 and spike antibodies were used to detect by immunoblotting. **d**The blocking capability of ground coffee and **e** instant coffee against SARS-CoV-2 entry was analyzed by cell-based TMPRSS2 enzyme activity assay. **f** Western blot was used to verify 293 T-ACE2 cells overexpressing TMPRSS2. **g** The impact of instant coffee tested by Vpp (MOI = 0.1) on TMPRSS2 involvement in SARS-CoV-2 entry in 293 T-ACE2 and 293 T-ACE2-TMPRSS2 cells. **h** Through Western blot to demonstrate coffee impacts cleavage of SARS-CoV-2 S by TMPRSS2. 293 T cells were transfected SARS-CoV-2 spike-HA and TMPRSS2-FLAG on 293 T cells for 6 h., then added with instant coffee for 24 h. **i** RT-qPCR was used to detect the regulation of the mRNA expression of ACE2 and TMPRSS2 on Huh7 (upper) and HepG2 (down) cells on instant coffee with 0, 0.25, 0.5, 1 mg/ml after 24 h. **j** Western blot to analyze the protein of ACE2 and TMPRSS2 on Huh7 and HepG2 treated with different doses of instant coffee after 24 h. **k** The quantification plots were analyzed from Western blot results. **l**, **m** The inhibition ability of instant coffee in different concentrations against wild-type SARS-CoV-2 on Huh7 and HepG2 cells was tested by Vpp (MOI = 0.1). **n** Huh7 cells were treated with different concentrations of instant coffee for CTSL activity, CL inhibitor as positive control. Statistical analyses were used by the Student T-test in triplicate independent experiments. Statistical significance was concerned as *P < 0.05, **P < 0.01 or ***P < 0.001

In accordance with the end of Fig. 1, we found that coffee can inhibit SARS-CoV-2 infection. Next, we addressed what mechanism is involved in coffee regulating the entry of SARS-CoV-2. The steps related to viral entry begin with the binding of the SARS-CoV-2 spike (S) to human ACE2 receptor [31]. First, we used an enzyme-linked immunosorbent assay (ELISA) to analyze whether ground and instant coffee against the spike protein-ACE2 binding. We observed that both ground coffee (IC50 = 4.87 mg/ml) and instant coffee (IC50 = 0.32 mg/ml) can interrupt the interaction of spike protein to ACE2 (Fig. 2a and b). Further, it was examined by a co-immunoprecipitation assay using 293T cells expressing SARS-CoV-2 spike-HA and ACE2. The result showed that instant (2, 4 mg/ml) can reduce the performance of pulldown ACE2 (Fig. 2c). The data demonstrated that coffee can inhibit the binding of ACE2 and spike. The mechanism that affects the entry of SARS-CoV-2 is not only through the binding of virus spike to host ACE2, but also depended on TMPRSS2 protease activity [32, 33]. To investigate whether coffee could arrest the entry of SARS-CoV-2 through impacting TMPRSS2 activity. We tested the effectiveness of ground and instant coffee for TMPRSS2 activity by a cell-based enzyme activity assay. The results showed that both ground (IC50 = 1.525 mg/ml) and instant coffee (IC50 = 0.136 mg/ml) can inhibit the activity of TMPRSS2 (Fig. 2d and e). Moreover, we transfected TMPRSS2 on 293T-ACE2 cells (Fig. 2f) to investigate the impact of coffee on TMPRSS2 activity and their relevance in SARS-CoV-2 entry by Vpp (Fig. 2g). The results showed that with the expression of TMPRSS2, coffee is able to suppress more effectively the SARS-CoV-2 entry. To investigate coffee whether impacts cleavage of SARS-CoV-2 S by TMPRSS2. As shown in Fig. 2h, furin is expressed naturally on 293T cells, when spike protein was expressed alone, the presence of both full-length S and S2 subunit which were cleaved by furin but not by TMPRSS2 and represent non-cleaved forma of spike protein for TMPRSS2 [34]. Co-expression of spike and TMPRSS2 led to a reduction in both S and S2 levels. In contrast, it was efficiently prevented by instant coffee at 2 mg/ml, resulting in a restoration of S and S2 expression. Therefore, the result obtained is that instant coffee can inhibit cleavage of SARS-CoV-2 S by TMPRSS2. Collectively, coffee has an activity to suppress SARS-CoV-2 entry, likely due to its potent activity to suppress SARS-CoV-2 S-ACE2 interaction and suppress TMPRSS2 effect to cleavage of SARS-CoV-2 S.

It was previously shown that SARS-CoV-2 may enter cells through the ACE2 receptor [35], and worth noting that the liver stands as a latent target for SARS-CoV-2 infection due to the detection of ACE2-positive cells within the liver [36–38]. Hepatocellular carcinoma (HCC) HepG2 cells revealed high ACE2 levels [39] and Huh7 expressed high TMPRSS2 [40] protein are suitable models for mechanistic research of SARS-CoV-2 entry. To analyze whether coffee can regulate the expression of ACE2 and TMPRSS2 on HCC cells to restrain the threat of SARS-CoV-2 infection. First, we treated different concentrations of coffee on Huh7 and HepG2 cells for 4 h., which significantly decreased the expression of ACE2 and TMPRSS2 genes as Fig. 2i. After 24 h., coffee was also found to lower the protein level of ACE2 and TMPRSS2 (Fig. 2j and k). Additionally, in the experiment of Vpp on Huh7 and HepG2 cells, it was observed that coffee at 1 mg/ml inhibits the entry of SARS-CoV-2 about 85% and 77%, respectively (Fig. 2l and m). During SARS-CoV-2 infection, in addition to TMPRSS2 but also cathepsin L (CTSL), facilitates SARS-CoV-2 entry [41, 42]. To identify whether coffee affects CTSL activity. Huh7 cells were treated with different concentrations of instant coffee for CTSL activity, as shown in Fig. 2n. The data indicated that instant coffee can significantly inhibit CTSL activity based on dose dependence and half maximal inhibitory concentration (IC50 = 0.37 mg/ml). Thus, the results suggested that coffee not only interrupts the binding of SARS-CoV-2 S to host ACE2 and reduces the activity of host TMPRSS2 and CTSL but also lowers the expression of ACE2 and TMPRSS2 on host cells, which helps to intensify the blocking of SARS-CoV-2 infection.

### Three isochlorogenic acids (isoCGAs) of coffee are recognized as more potent in inhibiting the entry of SARS-CoV-2

**Fig. 3:**
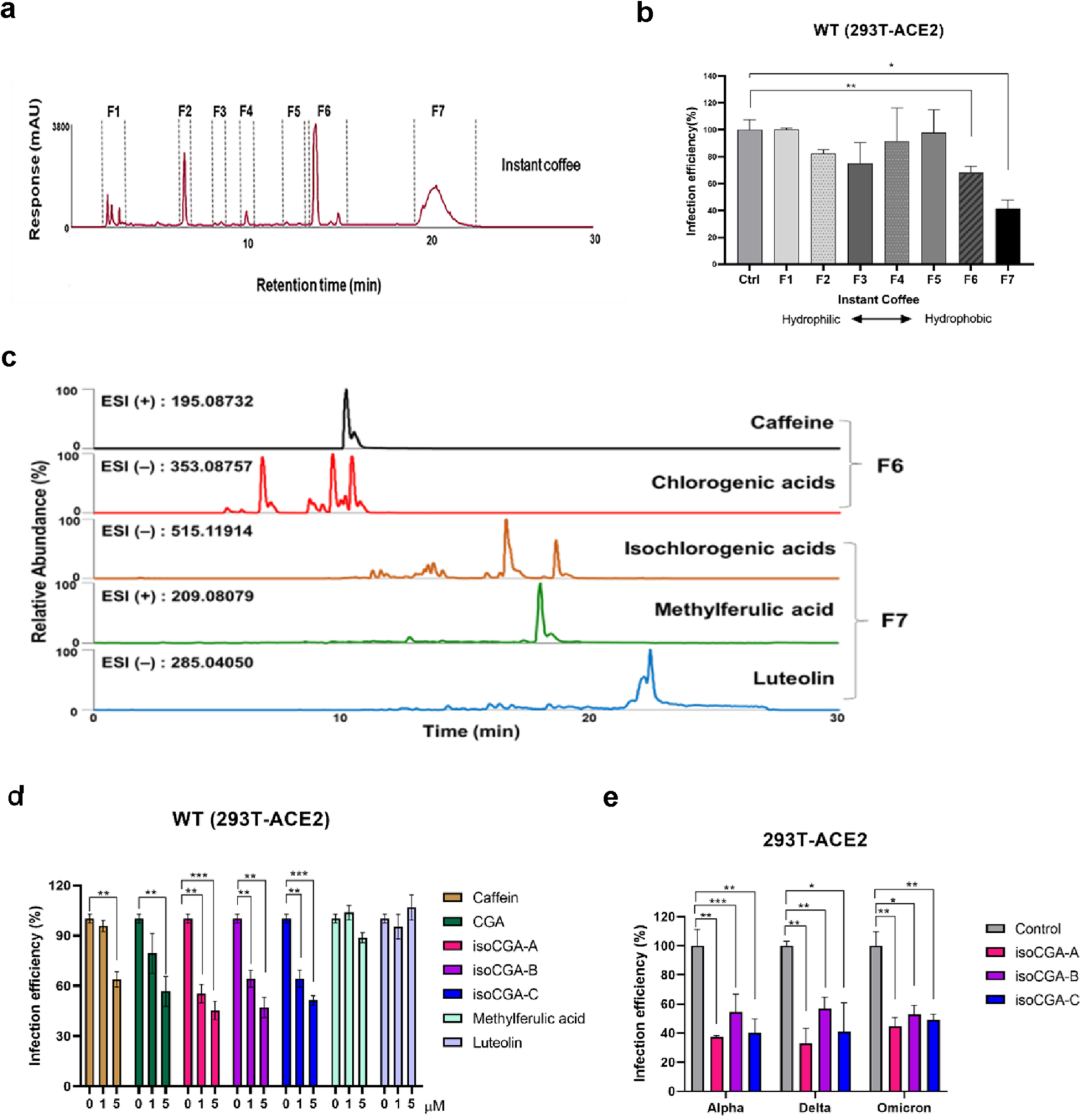
Hydrophobic compounds in coffee inhibit the SARS-CoV-2 pseudovirus entry to the cell. **a** Analysis of the main fractions of active compounds in coffee by UHPLC-HRMS analyses at 274 UV wavelengths (274 nm). **b** Wild-type SARS-CoV-2 Vpp (MOI = 0.1) was used to validate the inhibiting SARS-CoV-2 entry of 7 fractions from Hydrophilic to Hydrophobic on 293 T-ACE2 cells. **c** The extracted ion chromatograms (XICs) of instant coffee. Active compounds were detected including caffeine, chlorogenic acid (CGA); isochlorogenic acids (isoCGA-A, isoCGA-B and isoCGA-C), methylferulic acid and luteolin. **d** Examination of the inhibition capability of caffeine, CGA, isoCGA-A, isoCGA-B, isoCGA-C, methylferulic acid and luteolin against the entry of wild-type SARS-CoV-2 on 293 T-ACE2 cells by Vpp (MOI = 0.1). **e** Vpp (MOI = 0.1) was used to analyze the inhibition of isoCGA-A, isoCGA-B, and isoCGA-C with 5 μM separately against the entry of SARS-CoV-2 Alpha, Delta, and Omicron variants. *P* values were obtained by the Student T-test in triplicate independent experiments. Statistical significance was concerned as *P < 0.05, **P < 0.01 or ***P < 0.001

Next, we further analyzed which component may be involved in the capability to diminish SARS-CoV-2 infection. We used the method of "Here" (HRMS-exploring-recombination-examining) to identify the target compound of coffee. We analyzed instant coffee by UHPLC-HRMS analyses as shown in Fig. 3a, coffee expresses 7 main peaks at 274 UV wavelengths (274 nm). We collected these 7 separate fractions and applied Vpp to verify the ability to inhibit SARS-CoV-2 entry. It was found that fractions 6 and 7, which were biased towards hydrophobia, had a more potent inhibition on the entry of SARS-CoV-2 (Fig. 3b). Next, we used the databases to identify bioactive components in coffee and applied UHPLC-HRMS analyses to compare with the standard of pure compounds (Additional file 1: Figure S2a and Fig. 3c). The result revealed that the major organics in fraction 6 are caffeine and chlorogenic acid (CGA). The active compounds in fraction 7 are isochlorogenic acids as isochlorogenic acid A (isoCGA-A), isochlorogenic acid B (isoCGA-B) and isochlorogenic acid C (isoCGA-C), methylferulic acid and luteolin (Fig. 3c). Moreover, based on Fig. 3b, F7 fraction is more potent than F6 fraction in inhibiting SARS-CoV-2 entry, we wanted to understand whether the five compounds alone in the F7 fraction have the same effect as the F7 fraction in inhibiting the entry of SARS-CoV-2 pseudoviruses. First, we analyzed the content of 5 compounds of F7 fraction in coffee (Additional file 1: Figure S2b). On the basis of the proportion of the coffee ingredients from Additional file 1: Figure S2b, we tested the inhibitory of SARS-CoV-2 pseudovirus entry compared with the F7 fraction by each of the 5 compounds alone or reconstructing the mixture of the 5 compounds in a similar ratio of the F7 fraction (Additional file 1: Figure S2c). the results showed that when treated with a mixture of 5 compounds, the inhibition effect on SARS-CoV-2 pseudovirus entry was 33%, and the F7 fraction was 64%, indicating that the inhibitory effect of 5 compounds mixture is about half of F7 fraction. The results suggest there are some minor compounds that were not detected in the Fig. 3c but effective inhibitors which may be worthy of further study in the future. Next, we wondered whether these seven compounds found in F6 and F7 could repress the entry of SARS-CoV-2, respectively. From Vpp assay, we found that caffeine, CGA, isoCGA-A, isoCGA-B and isoCGA-C can remarkably suppress the entry of wild-type SARS-CoV-2, and the inhibition rate of isoCGA-A is the best, about 54%. In contrast, methylferulic acid and luteolin showed no inhibiton (Fig. 3d). Further, we tested the effects of isoCGA-A, isoCGA-B and isoCGA-C on other SARS-CoV-2 variants. The results presented that all three compounds could notably suppress the entry of SARS-CoV-2 pseudovirus, consistently isoCGA-A expressed a better inhibitory effect (Fig. 3e).

### Docking analysis also supports that isoCGAs more effectively compete with the spike-ACE2 protein interaction, and bind to the TMPRSS2 to avoid SARS-CoV-2 entry

**Fig. 4:**
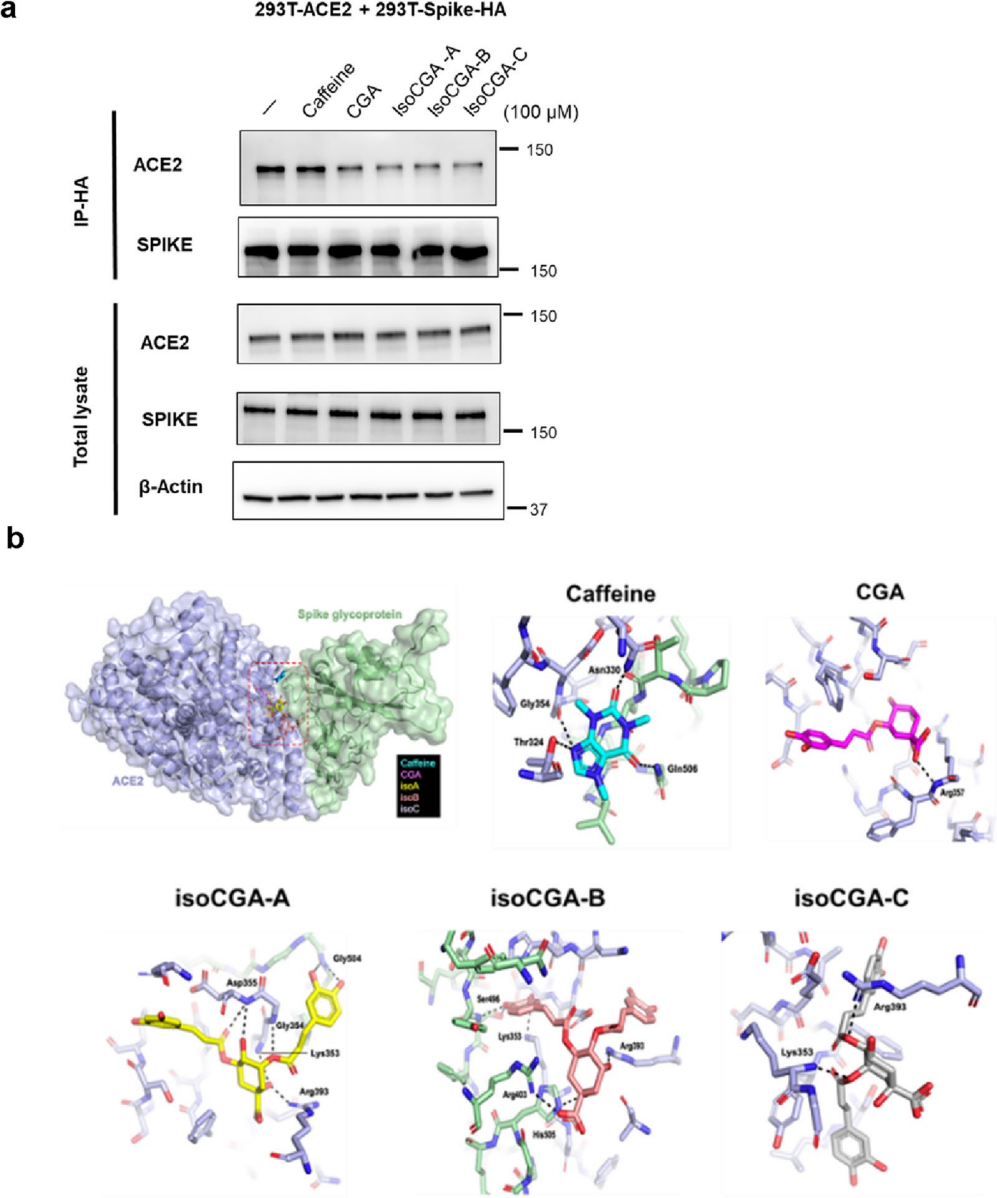
Molecular docking analysis with compounds of coffee and Omicron spike protein-ACE2 complex. **a** Co-immunoprecipitation using 293 T cells expressing SARS-CoV-2 spike-HA and ACE2 to verify that 5 compounds can inhibit the binding of ACE2 and spike. After anti-HA pulldown, adding 5 compounds (100 μM), ACE2, and spike antibodies were used to detect by immunoblotting. **b** Surface view of Caffeine, CGA, isoCGA-A, isoCGA-B and isoCGA-C docking to Omicron spike protein (S)-ACE2 complex (PDB ID: 7T9L). The best pose of each compound was shown as sticks. The binding area of compounds is indicated by red dashed rectangle. 3D visualization of hydrogen bond interactions between Caffeine, CGA, isoCGA-A, isoCGA-B and isoCGA-C with Omicron S-ACE2 complex amino acids. Four hydrogen bond interactions were formed between Caffeine and the complex amino acids Gln506 (S), Thr324 (ACE2), Asn330 (ACE2) and Gly354 (ACE2). One hydrogen bond interaction was formed between CGA and the complex amino acids Arg357 (ACE2). Seven hydrogen bond interactions were formed between isoCGA-A and the complex amino acids Gly504 (S), Lys353 (ACE2), Gly354 (ACE2), Asp355 (ACE2) and Arg393 (ACE2). Six hydrogen bond interactions were formed between isoCGA-B and the complex amino acids Arg403 (S), Ser496 (S), His505 (S), Lys353 (ACE2) and Arg393 (ACE2). Two hydrogen bond interactions were formed between isoCGA-C and the complex amino acids Lys353 (ACE2) and Arg393 (ACE2). Hydrogen bonds are shown as black dashed lines

We demonstrated that caffeine, CGA, isoCGA-A, isoCGA-B, and isoCGA-C can reduce SARS-CoV-2 infection. Additionally, we explored whether 5 compounds can block SARS-CoV-2 entry. First, we used a co-immunoprecipitation assay on 293 T cells expressing SARS-CoV-2 spike-HA and ACE2 to demonstrate. The result showed that CGA, isoCGA-A, isoCGA-B, and isoCGA-C (100 μM) can decrease the level of pulldown ACE2 (Fig. 4a). The data illustrated that CGA and isoCGAs can inhibit SARS-CoV-2 S-ACE2 interaction. To evaluate the interaction between these compounds and the spike protein-ACE2 complex at the atomic level, the molecular docking was performed by using the same program iGEMDOCK. On the basis of the docking score (Table 1), the top three compounds are isoCGA-C (−160.23 kcal/mol), isoCGA-A (−156.34 kcal/mol) and isoCGA-B (−152.19 kcal/mol). The fourth compound is CGA (−124.72 kcal/mol) and the last compound is caffeine (−79.51 kcal/mol). Interestingly, caffeine, CGA and isoCGA-B potentially binding to different binding sites (Fig. 4b and Additional file 1: Figure S3). Therefore, coffee might interrupt SARS-CoV-2 S-ACE2 binding through the synergy of these compounds.

**Table 1:** RBD/Ace2-binding energy scores calculated by iGEMDOCK 2.1

	Total energy (kcal/mol)	VDW (kcal/mol)	H Bond (kcal/mol)	Elec (kcal/mol)
isoCGA-C	−160.23	−132.18	−28.05	0.00
isoCGA-A	−156.34	−107.15	−47.52	−1.67
isoCGA-B	−152.19	−105.00	−43.31	−3.89
CGA	−124.72	−95.12	−29.89	0.30
Caffeine	−79.51	−59.74	−19.77	0.00

**Fig. 5:**
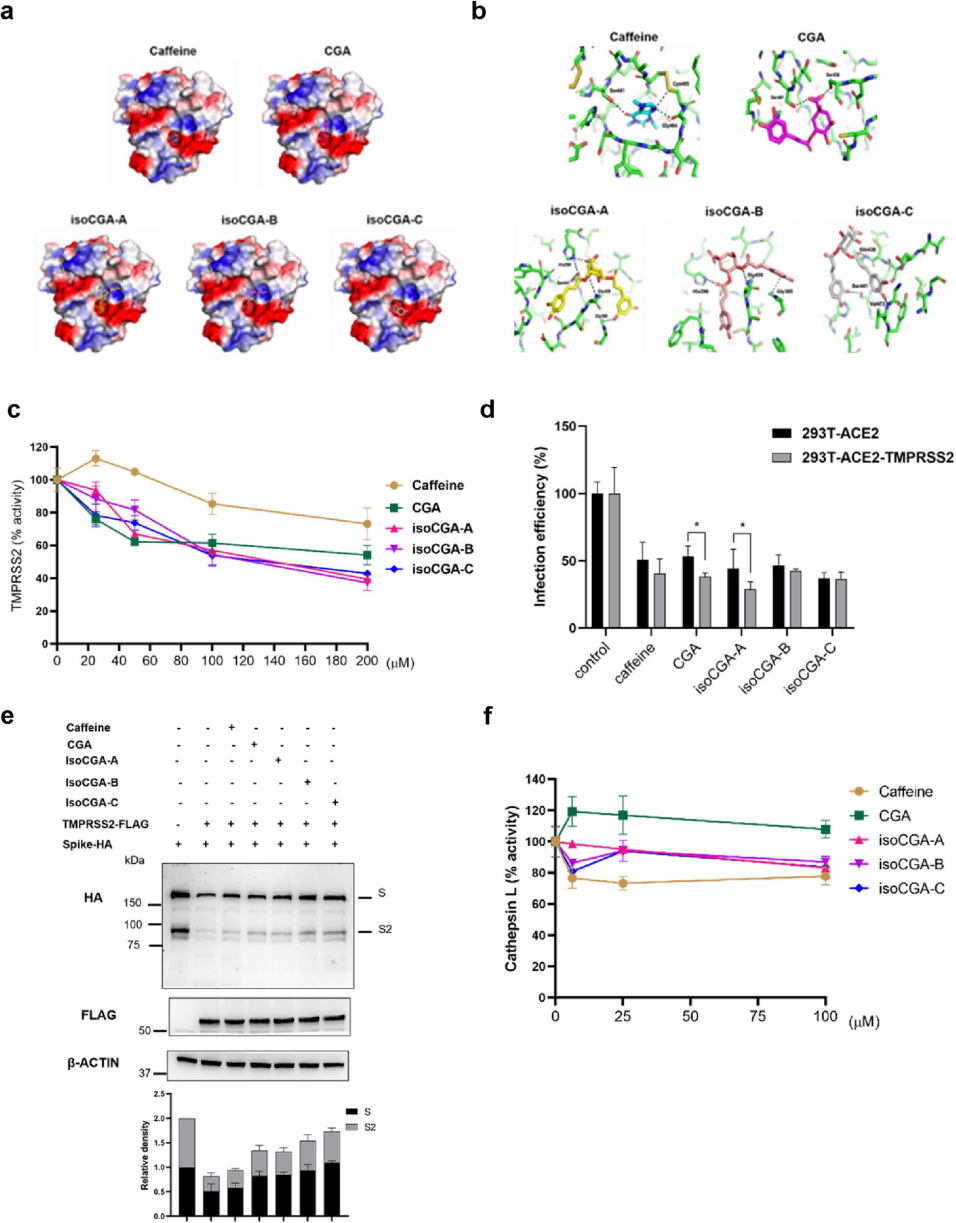
Molecular docking analysis with compounds of coffee and TMPRSS2. **a** Surface view of Caffeine, CGA, isoCGA-A, isoCGA-B and isoCGA-C docking to TMPRSS2. The surface charge distribution of TMPRSS2 shows the positively (blue) and negatively charged (red) area, respectively. **b** 3D visualization of hydrogen bonds between Caffeine, CGA, isoCGA-A, isoCGA-B and isoCGA-C with TMPRSS2 amino acids. Three hydrogen bond interactions were formed between Caffeine and TMPRSS2 amino acids Ser441, Gly464 and Cys465. Two hydrogen bond interactions were formed between CGA and TMPRSS2 amino acids Ser441 and Ser436. Five hydrogen bond interactions were formed between isoCGA-A and TMPRSS2 amino acids His296, Gly385, Ser441 and Gly439. Three hydrogen bond interactions were formed between isoCGA-B and TMPRSS2 amino acids His296, Gly385 and Gly439. Three hydrogen bond interactions were formed between isoCGA-C and TMPRSS2 amino acids Gln438, Ser441 and Val473. Hydrogen bonds are shown as black dashed lines. **c** Validation of the ability of caffeine, CGA, isoCGA-A, isoCGA-B, and isoCGA-C at 0, 25, 50, 100, and 200 μM to repress TMPRSS2 activity using a cell-based TMPRSS2 enzyme activity assay. **d** Effect of 5 compounds tested by Vpp (MOI = 0.1) on TMPRSS2 involvement in SARS-CoV-2 entry in 293 T-ACE2 and 293 T-ACE2-TMPRSS2 cells. **e** Through Western blot to test 5 compounds at 100 μM impact cleavage of SARS-CoV-2 S by TMPRSS2. **f** Huh7 cells were treated with different concentrations of 5 compounds at 0, 6.25, 25, and 100 μM for CTSL activity. Error bars indicated the standard error of the mean in triplicate independent experiments. *P* values were obtained by the Student T-test. Statistical significance was concerned as *P < 0.05

We also analyzed the interaction between these compounds and the TMPRSS2 at the atomic level, the molecular docking was performed by using the iGEMDOCK v2.1. The best pose of each compound docking to TMPRSS2 was predicted (Fig. 5a, b and Additional file 1: Figure S4) and the binding energy values (docking scores) of the compounds and TMPRSS2 are rated (Table 2). Based on the docking score, the top three compounds are isoCGA-B (−155.23 kcal/mol), isoCGA-A (−154.02 kcal/mol) and isoCGA-C (−153.75 kcal/mol). The fourth compound is CGA (−127.06 kcal/mol) and the last compound is caffeine (−90.91 kcal/mol). Notably, all of these five compounds utilize hydrogen bond interactions with the canonical Ser441-His296-Asp345 catalytic triad in this trypsin-like serine peptidase domain. Caffeine, CGA, isoCGA-A and isoCGA-C have hydrogen bond interactions with Ser441; isoCGA-A and isoCGA-B have hydrogen bond interactions with His296. This docking result provides a structural basis for the direct inhibition of the TMPRSS2 by caffeine, CGA and isoCGAs. Moreover, we tested the effect of caffeine, CGA, and isoCGAs on TMPRSS2 activity. The data revealed that isoCGA-A, isoCGA-B and isoCGA-C can inhibit the activity of TMPRSS2 better than caffeine and CGA (Fig. 5c). The result is consistent to the molecular docking. Furthermore, we demonstrated the role of 5 compounds on TMPRSS2 activity and their relation to SARS-CoV-2 entry by Vpp in 293 T-ACE2-TMPRSS2 cells. It showed that CGA and isoCGA-A can inhibit the SARS-CoV-2 entry notably with the expression of TMPRSS2 (Fig. 5d). Additionally, we tested the cleavage of SARS-CoV-2 S through TMPRSS2 under treatment with 5 compounds. As shown in Fig. 5e, S and Additional file 1: Figure S2 cleavage were prevented by CGA and isoCGAs at 100 μM. Therefore, the result obtained is that CGA and isoCGAs can depress the cleavage of SARS-CoV-2 S by TMPRSS2. To test whether 5 compounds affect CTSL activity. We treated 5 compounds at indicated concentrations in Huh7 cells for analysis of CTSL activity, as shown in Fig. 5f. These results indicated that 5 compounds have no significant inhibition of the activity of CTSL, indicating that other ingredients of coffee may have the potential to suppress the activity of CTSL and are worthy of further research. The above results from coffee analyses, in silico and in vitro experiments, demonstrated that coffee has the ability to suppress SARS-CoV-2 entry owing to a variety of bioactive compounds. Compared with caffeine, CGA, and isoCGAs have a better ability to block SARS-CoV-2 infection.

**Table 2:** TMPRSS2-binding energy scores calculated by iGEMDOCK 2.1

	Total energy (kcal/mol)	VDW (kcal/mol)	H Bond (kcal/mol)	Elec (kcal/mol)
isoCGA-B	−155.23	−115.84	−37.46	−1.93
isoCGA-A	−154.02	−109.91	−42.09	−2.02
isoCGA-C	−153.75	−105.79	−48.21	0.24
CGA	−127.06	−84.61	−42.53	0.08
Caffeine	−90.91	−62.92	−27.99	0.00

### Decaffeinated coffee remains effective against SARS-CoV-2 infection

**Fig. 6:**
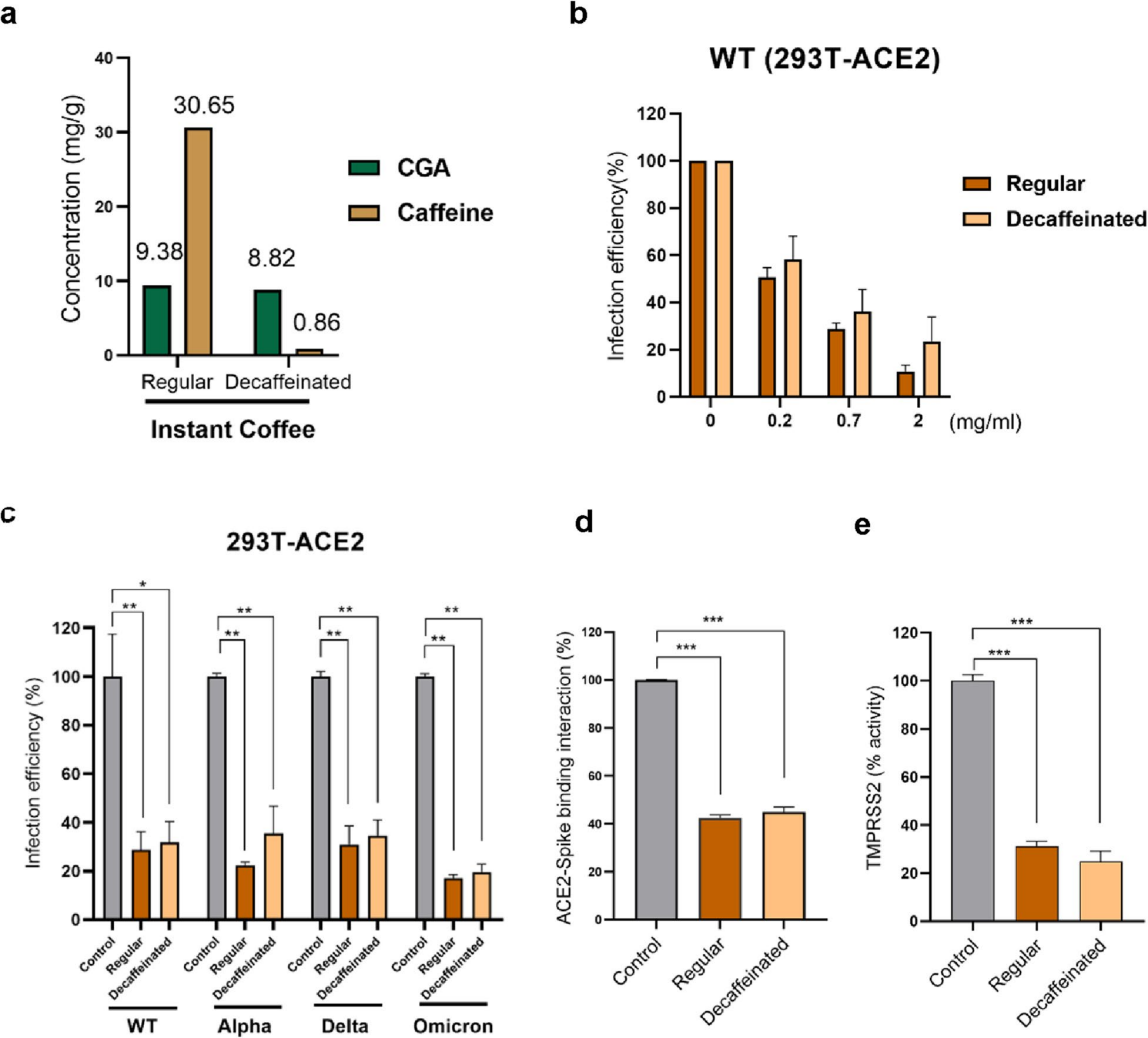
Decaffeinated coffee still has the ability to block SARS-CoV-2 variants infection. **a** HPLC analysis was used to examine the caffeine and CGA content of commercially available decaffeinated coffee. **b** By Vpp (MOI = 0.1), regular and decaffeinated coffee were verified to reduce the entry of wild-type SARS-CoV-2 on 293 T-ACE2 cells. **c** Through Vpp (MOI = 0.1), regular and decaffeinated coffee were examined to inhibit the entry of wild-type, Alpha, Delta, and Omicron variants on 293 T- ACE2 cells. **d** Regular and decaffeinated coffee were analyzed for the effect of blocking ACE2/spike interaction by Enzyme-linked immunosorbent assay. **e** Regular and decaffeinated coffee were examined to decrease TMPRSS2 activity by cell-based TMPRSS2 enzyme activity assay. Error bars indicated the standard error of the mean in triplicate independent experiments. *P* values were obtained by the Student T-test. Statistical significance was concerned as *P < 0.05, **P < 0.01 or ***P < 0.001

Caffeine is a methylxanthine that antagonizes sleep-regulating adenosine A1 and A2A receptors, lengthening sleep latency and reducing total sleep time [43, 44]. According to previous reports, long-term or excessive caffeine consumption can cause insomnia, migraine, anxiety, and animation. Therefore, the elderly, adolescents, pregnant women, and caffeine-sensitive people are susceptible to the effects of caffeine intake [45–47]. For the above reasons, we attempted to test whether decaffeinated coffee could still preserve the efficacy of inhibiting the infection with SARS-CoV-2. First, we obtained commercial decaffeinated coffee from supermarkets and verified whether it was decaffeinated by UHPLC-HRMS analyses. The results clarified that decaffeinated coffee contains nearly no caffeine and retains the CGA content in coffee (Fig. 6a). Next, we used Vpp to test the capability of decaffeinated coffee to reduce the entry of SARS-CoV-2. Compared with regular coffee, it still has the ability to inhibit the entry of SARS-CoV-2 (Fig. 6b) and also presents a significant effect on reducing the entry of SAR-CoV-2 variants (inhibition ability 64% to 80%) as shown in Fig. 6c, i.e. without caffeine, it still inhibits SARS-CoV-2 infection. Moreover, we also examined whether decaffeinated coffee has the ability to block spike-ACE2 binding and inhibit TMPRSS2 activity. We found that decaffeinated coffee also significantly decreased the 55.8% interaction between spike protein and ACE2 receptor and reduced 75.1% TMPRSS2 activity (Fig. 6d and e). Thus, decaffeinated coffee still has the capability to interrupt SARS-CoV-2 infection, which is important to know for caffeine-sensitive populations to drink decaffeinated coffee to prevent SARS-CoV-2 infection.

### Serum from humans who drink coffee exist the capability to suppress the SARS-CoV-2 infection

**Fig. 7:**
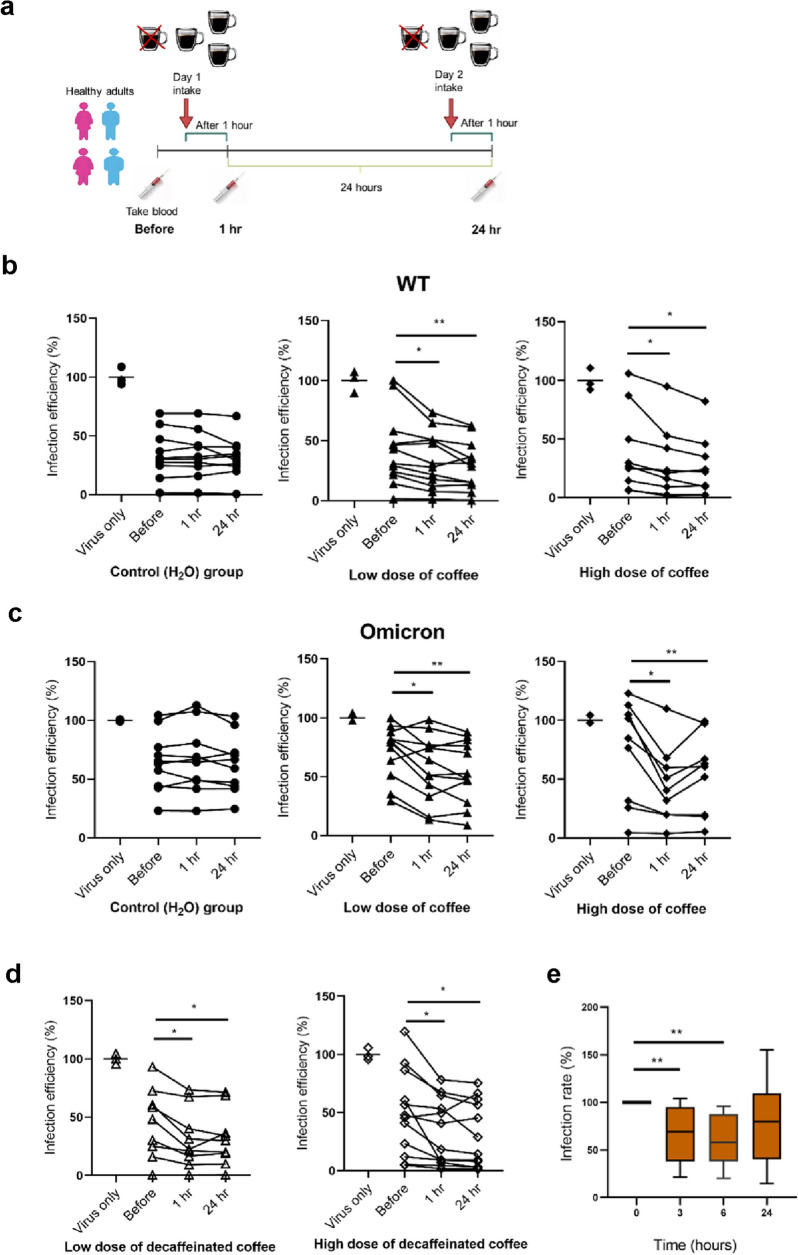
Serum from drinking coffee samples has an inhibitory effect against SARS-CoV-2 variants. **a** Flow chart of collecting serum within 24 h. in coffee-drinking subjects. **b** Detection of inhibitory efficacy of the serum with control group (H_2_O), low dose of coffee (1 cup), and high dose of coffee (2 cups) against wild-type SARS-CoV-2 by Vpp. **c** The inhibiting capability of the serum in drinking coffee with different concentrations against the Omicron variant was assessed by Vpp. **d** Efficacy against the Omicron variant of the serum with a low dose of decaffeinated coffee (1 cup), and a high dose of decaffeinated coffee (2 cups) was tested by Vpp. **e** Vpp assay examined that serum reveals the ability to inhibit the SARS-CoV-2 Omicron variant infection within 6 h. after drinking coffee. SARS-CoV-2 Vpp assay (MOI = 0.1). Analysis of variance (ANOVA) was performed for statistical analyses. Statistical significance was concerned as *P < 0.05, **P < 0.01 or ***, *P* < 0.001

The above results prompted us to test in people who drink coffee whether their serum may be against the entry of SARS-CoV-2 (Additional file 1: Figure S5a). We accumulated a human trial of 64 healthy participants from Taiwan. The entire trial had a median age of 29.3 years (range: 21–40 years) and 47% were female (Table 3). First, the participants were randomly assigned to a control group (water), low-dose regular coffee (4 mg, approximate 1 cup/ 240 ml), high-dose regular coffee (8 mg, approximate 2 cups), and high-dose decaffeinated coffee (8 mg, approximate 2 cups). Before intake of coffee, the first blood was taken to obtain serum (Before). Next, intake on the basis of the group classification, second blood was performed 1 h. later (1h). After 24 h., participants drink water or coffee again and had a third blood draw 1 h. later (24h). The detailed experimental procedure is shown in Fig. 7a. Sera from the trial were executed with Vpp to assess whether coffee intake acts as a booster to inhibit infection of the SARS-CoV-2 variants. Before the Vpp experiment, the collected serum was examined by HPLC analyses for the metabolism of caffeine in vivo since caffeine was routinely used as a marker for coffee drinking [48]. From the result in Additional file 1: Figure S5b, we understood that the caffeine of serum in the high- concentration group is approximately twice that of the low-concentration group, and this data matched the concentration multiples of coffee drinking in our designed experiment. In the results of the Vpp experiment, the enhancing inhibition of wild-type and SARS-CoV-2 variants including the Omicron variant could be detected obviously in most of the individuals drinking coffee containing low and high concentrations (Fig. 7b, c, and Additional file 1: Figure S5c and d). Therefore, drinking more than 1 cup of coffee a day can effectively reduce SARS-CoV-2 infection.

**Table 3:** Subject demographic information

Characteristic	Control group (n = 10)	Coffee group (n = 33)	Decaf coffee group (n = 21)
Sex-No. (%)			
Male	3 (30)	16 (48.5)	15 (71.4)
Female	7 (70)	17 (51.5)	6 (28.6)
Age (years)	32.1 ± 5.3	28.8. ± 4.6	29.0 ± 5.3
Weight (kg)	61.1 ± 12.3	66.8 ± 13.6	63.2 ± 18.2
Vaccination status-No. (%)			
1 doses	0 (0)	0 (0)	1 (4.8)
2 dose	0 (0)	3 (9.1)	0 (0)
3 doses	10 (100)	29 (87.9)	20 (95.2)
4 doses	0 (0)	1 (3)	0 (0)
Covid-19 infection-No. (%)			
Not infected	10 (100)	33 (100)	21 (100)
Infected	0 (0)	0 (0)	0 (0)

Data are given as mean ± SD. The detailed vaccination status of each subject is shown as Additional file 2: Table S1

Moreover, in the decaffeinated coffee test, it was shown that even drinking decaffeinated coffee can effectively reduce SARS-CoV-2 entry, especially in the high-dose decaffeinated group (Fig. 7d). In this human trial experiment, we also verified the timeliness of effectively inhibiting SARS-CoV-2 entry after drinking coffee (from 0 to 24 h.). The results of HPLC analysis showed that the caffeine in serum reached the highest concentration after 3 h. of coffee drinking, and then decreased with time until the lowest concentration of caffeine in serum was obtained after 24 h. (Additional file 1: Figure S5e). Moreover, the effect of inhibiting SARS-CoV-2 entry was tested by Vpp assay. Figure 7e showed that after drinking coffee, the entry of the Omicron variant pseudovirus can be significantly inhibited within 6 h. The data suggested that the optimal timeline for coffee to inhibit SARS-CoV-2 infection is within 6 h. Taken together, drinking 1–2 cups of coffee as well as decaffeinated coffee daily can potently reduce SARS-CoV-2 infection including wild-type, Alpha, Delta, and Omicron variants, which can serve as a guideline for dietary health during coexistence with SARS-CoV-2.

## Discussion

With the outbreak of COVID-19 at the end of 2019, scholars around the world devoted themselves to solving the pressing difficulties. Vu et al. analyzed the data from the UK Biobank to find that coffee intake was associated with lower positives for COVID-19 [26]. However, further studies have yet to demonstrate the exact possible mechanism that coffee impedes the binding of SARS-CoV-2 to host cells. Our results demonstrated that coffee, a beverage readily available, can be a new strategy to reduce SARS-CoV-2 infection via blocking spike protein- ACE2 interaction, inhibiting TMPRSS2 and CTSL activity, and diminishing the protein level of TMPRSS2 and ACE2, respectively. Interestingly, no matter what kind of coffee or adding additional intergrading (sugar or milk), it has the capability to reduce SARS-CoV-2 entering into host cells. Our research supported drinking coffee is related to reducing COVID-19 infection, which is consistent with the opinion of meta-analyses that reported coffee consumption related to a lower COVID-19 positive probability.

Further, we isolated coffee into 7 fractions by UHPLC -HRMS. It was found that fractions 6 and 7 had better efficacy against SARS-CoV-2. The main bioactive compounds of fraction 6 contained caffeine and CGA, and fraction 7 included isochlorogenic acid A (isoCGA-A), isochlorogenic acid B (isoCGA-B) and isochlorogenic acid C (isoCGA-C), methylferulic acid and luteolin. Some articles mentioned that caffeine using molecular docking models predicted that the virus might infect host cells by inhibiting the binding of the SARS-CoV-2 spike with ACE2 [49, 50]. CGA may have the ability to inhibit SARS-CoV-2 entry based on its powerful polyphenol antioxidant [51]. We not only demonstrated that caffeine and CGA were able to reduce the entry of SARS-CoV-2 but also found that isochlorogenic acids presented stronger binding to spike-ACE2 protein than CGA and caffeine for the omicron variant by molecular docking mode. Moreover, molecular docking also showed that isochlorogenic acids similarly have the strongest affinity to bind to the TMPRSS2 protein. And, we verified that isochlorogenic acids had the strongest inhibitory effect on TMPRSS2 activity. In addition, recent studies pointed out that isoCGA-A has a strong binding to Mpro protein by molecular docking, and remdesivir, an antiviral compound, has the same effect [52]. Therefore, isochlorogenic acids would be the crucial phytochemicals that exist in coffee to reduce the infection with multiple variants of SARS-CoV-2.

Most of the previous publications about coffee and COVID-19 were review articles or clinical statistical analyses [26, 27, 51]. Differently, in our human trial, we collected sera from non-drinking coffee, low-dose, high-dose, and decaffeinated coffee participants, and verified by high-throughput Vpp that coffee intake participants' sera had enhanced the response to neutralization of SARS-CoV-2. As expected, the sera of coffee-drinking participants including the decaffeinated group were observed to increase potency in neutralizing wild-type and alpha variant SARS-CoV-2. However, for the Omicron variant, the previous articles pointed out that the Omicron variant has more mutation points and a higher ability of immune escape than other variants [4, 53]. Therefore, many strategies may be ineffective or have poor responses to keep off the infection from the Omicron variant [54, 55]. Interestingly, it was shown that drinking coffee is beneficial to achieve inhibitory potency to inhibit the SARS-CoV-2 variants in our study. In addition, we showed that the best timeline for impeding SARS-CoV-2 entry is within 6 h. after drinking coffee. It is recommended to intake coffee once again after 6 h. to maintain the efficacy of inhibiting SARS-CoV-2 infection.

## Conclusions

**Fig. 8:**
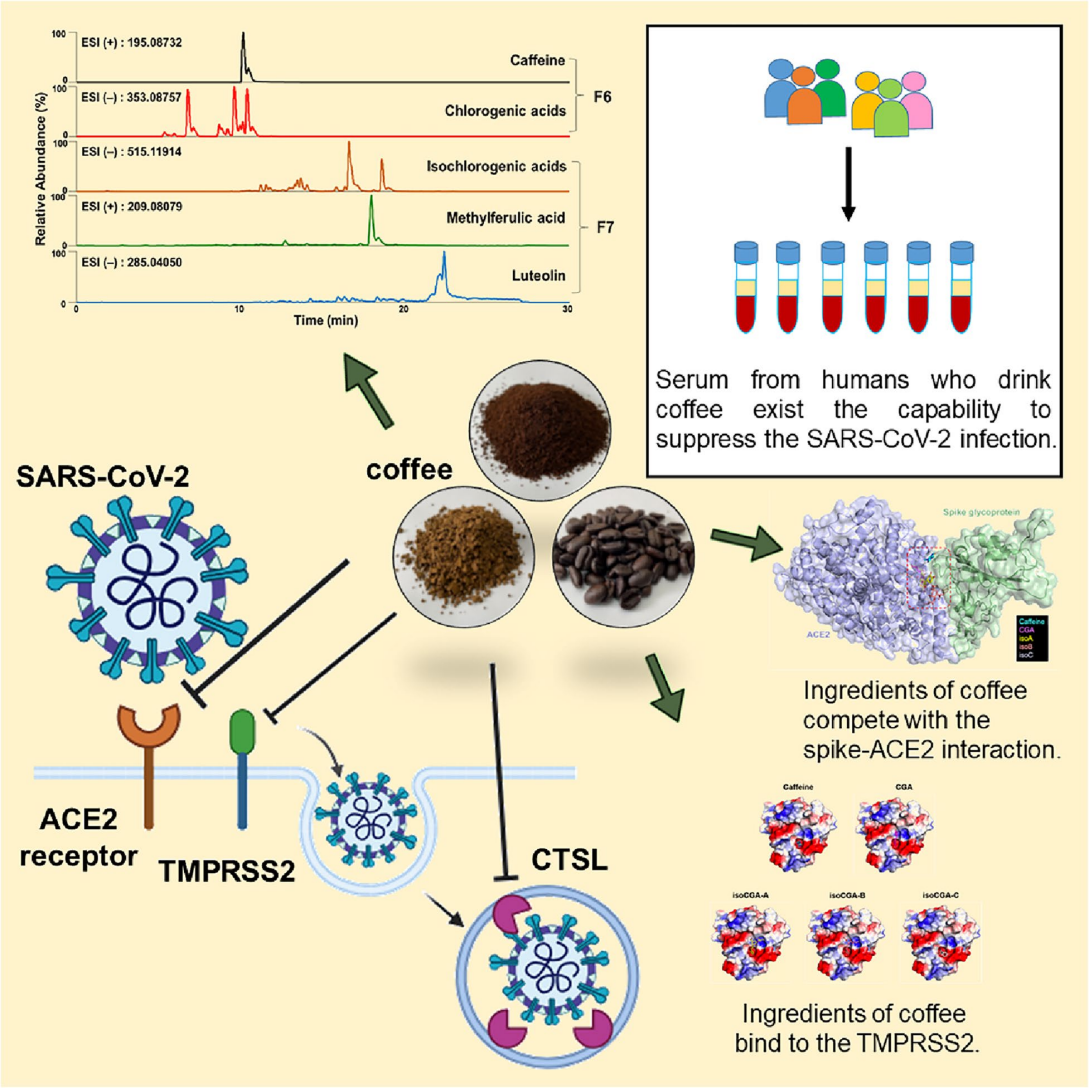
Schematics of the model of coffee in preventing SARS-CoV-2 infection. Exploring potential compounds in coffee against SARS-CoV-2 infection, verifying the mechanism of suppressing SARS-CoV-2 entry, and identifying coffee to prevent SARS-CoV-2 infection in human trials. The model was created by bioRENDER

Taken together, our in vitro identified that coffee can inhibit SARS-CoV-2 infection through the mechanism of blocking spike protein- ACE2 interaction, inhibiting TMPRSS2 and CTSL activity, and decreasing the protein level of TMPRSS2 and ACE2 on host cells, respectively (Fig. 8). And, our human trial study suggested that drinking coffee (approximately 1–2 cups per day) has a potentiated ability to suppress the infection with present SARS-CoV-2 strains (including the Omicron variant), and proposed that drinking coffee can be adopted in the new COVID-19 protection guide to limit the risk of infection with SARS-CoV-2.

## Research design and methods

### Material

Various categories of ground coffee (Laos, Honduras, Indonesia, Guatemala, and USA), instant coffee (Japan, Brazil and Germany), decaffeinated coffee (Japan), and additives including creamer, low-fat milk, sugar, and liquid sugar were obtained from supermarkets. Briefly, 9 g coffee beans were ground and soaked in 150 mL of hot water (95 °C) in a cup for three minutes. 3 g instant powder was dissolved in 150 mL of hot water (95 °C) in a cup. Ground and instant coffee were centrifuged at 1,000 rpm for 5 min, and the supernatant was filtrated by a 0.22 μm filter. The filtrated ground and instant were as stock and stored at − 80 °C. The standard pure compounds were purchased including caffeine, and chlorogenic acid (Sigma-Aldrich), Methylferulic acid and luteolin (Nature Standard), Isochlorogenic acid A, B, and C (MedChemExpress).

### Cell culture

For transient transfections, HEK293T cells were seeded and pCMV3-ACE2 plasmid, pCMV3-TMPRSS2-FLAG (Sino Biology) and pcDNA3.1( +) − 2019-nCoV-S (Wuhan strain wild type, RNA Technology Platform and Gene Manipulation Core, Academia Sinica, Taiwan) were transfected. Transfected cells cultured in DMEM containing 10% FBS, and 1% penicillin/streptomycin (P/S). More contents were referred in references [56]. Non-small-cell lung cancer cell line NCI-H460 was maintained in MEM added with 10% FBS, and 1% P/S. Human hepatoma Huh7 and HepG2 cell lines were cultured in DMEM containing 10% FBS and 1% P/S. All cells were cultured at 37 °C/5% CO_2_.

### SARS-CoV-2 viral pseudo-particle (Vpp) infection assay

Variants strain SARS-CoV-2 pseudoparticles were purchased from RNAi core, Academia Sinica, Taiwan (http://rnai.genmed.sinica.edu.tw/). 1 × 10^4^ (293T-ACE2), and 5 × 10^3^ (NCI-H460) cells were seeded into 96-well plates, and pretreated with varying doses of the indicated sample for 1 h., then infected pseudovirions (MOI = 0.1). After 24 h. incubation, cell viability was measured by Cell Counting Kit-8 (Dojindo Laboratories). Next, every sample was added Bright-Glo Luciferase Detection System (Promega), and the luminescence detected through the GloMax Navigator System (Promega). More contents were referred in references [57].

### ELISA for spike-ACE2 binding

The spike-ACE2 Binding Assay Kit II (RayBiotech Life) is an enzyme-linked immunosorbent assay, was applied to examine the ground, instant, and decaffeinated coffees neutralized SARS-CoV-2 spike binding with host cell ACE2 protein obeying the reference [58].

### Cell-based enzyme activity assay for TMPRSS2

1.5 × 10^4^ Huh7 cells were seeded in a 96-well plate. After 24 h., 80 μL of PBS, ground, instant coffee, caffeine, CGA and isoCGAs respectively in the indicated concentrations were substituted for the medium and incubated at room temperature for 1 h. The Boc-QAR-AMC substrate (R&D Biosystems) was used at the final concentration 100 μM in a total of 100 μL reaction mixture. Fluorescence (excitation/emission peaks at 380/460 nm) was monitored using Synergy^™^ H1 hybrid multi-mode microplate reader (BioTek Instruments, Inc.). More contents were referred in references [59].

### Analysis of cathepsin L (CTSL) activity

1 × 10^6^ cells Huh7 cells were obtained and lysed cells in 50 µL. Centrifuge the sample for 5 min at 4 °C and collect the supernatant in a clean tube. Adding instant coffee, caffeine, CGA, and isoCGAs separately in the indicated concentrations. The Cathepsin L Assay Kit (Novus Biologicals) was used at the final concentration 200 μM in a total of 100 μL reaction mixture. Mix and incubate at 37 °C for 2 h. Fluorescence (excitation/emission peaks at 400/505 nm) was monitored using Synergy^™^ H1 hybrid multi-mode microplate reader (BioTek Instruments, Inc.).

### Molecular docking (TMPRSS2, spike/ACE2, and spike protein)

The TMPRSS2 crystal structure (PDB ID: 7MEQ) and Omicron spike protein-ACE2 complex (PDB ID: 7T9L) were prepared using PyMOL Molecular Graphics System, Version 2.0 Schrödinger, LL. We removed all water molecules and bound ions. The binding location of the ligand 4-carbamimidamidobenzoic acid (GBS) in the TMPRSS2 structure was considered as the active site. The amino acids residues surrounding the GBS within 8 Å were defined as the binding site residues. To define the binding site of the spike protein-ACE2 complex [60], we analyzed the interface of the complex using PISA [61] and selected the binding site amino acids manually for the docking process. Compounds were downloaded from the PubChem database (https://pubchem.ncbi.nlm.nih.gov/ accessed on 9 December 2022) as a SDF file and converted into MOL file using Online SMILES Translator and Structure File Generator (https://cactus.nci.nih.gov/translate/ accessed on 9 December 2022). Docking tasks were performed using iGEMDOCK [62] with parameters: Population size = 300, Generation = 80 and Number of solutions = 100. The best docking pose with the lowest energy value (docking score) was selected for further interaction analysis. All the docking results visualization was represented using PyMOL and LigPlot + v.2.2.5 [63].

### UHPLC-HRMS analyses

The UHPLC-HRMS analyses were performed on the Ultimate 3000 UHPLC coupled to Q-Exactive Plus mass spectrometer (Thermo Scientific). The heated electrospray ionization (H-ESI) source with positive and negative ionization modes was used to ionize samples. The UHPLC separation was performed by using Waters ACQUITY UPLC C18 column (100 × 2.1 mm i.d., 1.7 μm, Waters). Mobile phase A and B used in this study were 0.2% formic acid aqueous solution and methanol, respectively. The gradient used in UHPLC separation was as follows: 0–1.5 min, 5% B; 1.5–20 min, 5–50% B; 20–20.1 min, 50–90% B; 20.1–25 min, 90% B; 25–25.3 min, 90–5% B; 25.3–30 min, 5% B. The full scan-data dependent acquisition (MS/ddMS2) scan (full MS/ddMS2) mode was used for qualification and quantification of ingredients in coffee. The resolution power for full-MS and ddMS2 were set for 70,000 and 35,000, respectively. The Compound Discoverer software (Thermo Scientific) with using ChemSpider and mzCloud databases was used to processes HRMS data and identify bioactive components in coffee.

### HPLC analyses

The Agilent 1260 Infinity II HPLC system (Agilent) was utilized for fraction collection of coffee ingredients and quantification of caffeine in serum. The chromatographic separation was performed using Agilent Poroshell C18 column (4.6 × 150 mm i.d., 4 μm). For fraction collection, the mobile phase A and B were 0.2% formic acid aqueous solution and acetonitrile and the gradient was as follows: 0–16 min, 5–15% B; 16–20 min, 15–70% B; 20–25 min, 70% B; 25–26 min, 70–5% B; 26–30 min, 5% B. The flow rate was 0.7 mL/min and the injection volume was 100 μL. The wavelength of 274 nm was utilized for detection of ingredients in coffee. For analysis of caffeine in serum, the water containing 5 mM ammonium acetate as well 0.1% formic acid and methanol were used as mobile phase A and B, respectively. The gradient was as follows: 0–3 min, 5–30% B; 3–10 min, 30% B; 10–15 min, 30–95% B; 15–18 min, 95% B; 18–19 min, 95–5% B; 19–22.5 min, 5% B. The column temperature was 40 ℃ as well as flow rate was 0.4 mL/min. The caffeine was detected under the wavelength of 280 nm. The protein precipitation was used for preparing the serum sample in this study and the procedure was as follow: 100 μL serum sample and 250 μL acetonitrile which was containing 0.1% formic acid were placed in a 1.5 mL centrifuging tube and then vortexed for 5 min. After centrifuging for 15 min at 4 ℃, a 250 μL supernatant was transferred to a clean 1.5 mL centrifuging tube and dried by using a CentiVap Concentrator (LABCONCO). The residue was dissolved by 100 μL water containing 0.2% formic acid-acetonitrile (1:1, v/v). After vortex for 1 min then centrifuging for 5 min, a 10 μL supernatant was injected onto HPLC for analysis.

### Real-time reverse transcription (RT)-PCR

Total RNA was isolated with TRIzol reagent (Invitrogen) on the basis of the manufacturer’s instructions. The cDNA was synthesized by M-MLV reverse transcriptase (Promega). RT-PCR was performed by GoTaq qPCR Master Mix (Promega) and the Applied Biosystems StepOnePlus^™^ System to quantify the gene expression. The mRNA expression was normalized to GAPDH. The primer information is shown in Table 4.

**Table 4:** Primers used in this study

Gene	Forward primer (5ʹ–3ʹ)	Reverse primer (5ʹ–3ʹ)
ACE2	TCCATTGGTCTTCTGTCACCCG	AGACCATCCACCTCCACTTCTC
TMPRSS2	TAGTGTCCCCAGCCTACCTC	GCACCAAGGGCACTGTCTAT
GAPDH	ATCACCATCTTCCAGGAGCGA	AGCCTTCTCCATGGTGGTGAA

### Co-immunoprecipitation

For immunoprecipitation, lysates of 293 T-SARS Cov2 spike-HA cells were incubated with anti-HA magnetic beads (Thermo Scientific) for 30 min at 4 °C. Add coffee or compounds to lysates for 1 h at 4 °C. Then 293 T-ACE2 lyate was added overnight, 4 °C. The protein bead mixture was collected and washed three times at 4 °C. The immunoprecipitants were subjected to SDS-PAGE gel and Western blot. The primary antibodies were anti-SARS-CoV-2 spike glycoprotein antibody (Abcam), anti-ACE2 (Cell Signaling) and anti-Actin (Santa Cruz Biotechnology). Input controls of full-length spike protein after anti-HA pulldown from cell lysates mixed adding instant coffee to affect the binding of ACE2 and spike, then ACE2 and spike antibodies were used to detect.

### Western blot assay

Equal amounts of protein were run on SDS-PAGE gel and blotted onto a PVDF membrane. The primary antibodies incubated at 4 ℃ overnight were anti-ACE2 (Cell Signaling), anti-TMPRSS2 (Santa Cruz Biotechnology), and anti-Actin (Santa Cruz Biotechnology). Immunoreactive bands were detected with the ECL Substarte (BIO-RAD) through a iBright Imaging Systems (Thermo Fisher).

### Human subjects

The application of human samples was approved by Research Ethics Committee China Medical University & Hospital, Taichung, Taiwan (CMUH111-REC3-093). All participants provided written informed consent before enrollment. Serum samples from 64 participants were collected. For each individual information including age, sex, weight, COVID-19 vaccine, and any related medical history was received.

### Human serum SARS-CoV-2 Vpp assay

SARS-CoV-2 Vpp were generated based on the VSV packaging system with different spike proteins and luciferase reporter genes to compare the neutralizing activity of participants' serum against SARS-CoV-2. Each serum sample was diluted 1:200 and pretreated into 293T-ACE2 cells for 1 h., subsequently, added pseudovirions (MOI = 0.1) for 24 h. [64]. Next, the following experimental procedures and measurements acted in accordance with the SARS-CoV-2 Vpp infection assay.

### Statistical analysis

Data analyses were presented by GraphPad Prism 8. Analysis of variance (ANOVA) and Student T-test was performed for statistical analyses. Error bars indicated the standard error of the mean in triplicate independent experiments. Statistical significance was concerned as *P < 0.05, **P < 0.01 or ***P < 0.001.

### Supplementary Information

**Additional file 1: Figure S1.** Identification of cell viability. **Figure S2.** Evaluation of the content of compounds in coffee and the inhibiting efficacy against SARS-CoV-2 entry. **Figure S3.** Ligplot + shows the interactions between Caffeine, CGA, isoCGA-A, isoCGA-B and isoCGA-C with Omicron SP-ACE2 complex amino acids. Hydrogen bonds are shown as green dashed lines. Hydrophobic contacts are represented as spline curves. **Figure S4.** Ligplot + shows the interactions between Caffeine, CGA, isoCGA-A, isoCGA-B and isoCGA-C with TMPRSS2 amino acids. Hydrogen bonds are shown as green dashed lines. Hydrophobic contacts are represented as spline curves. **Figure S5.** Drinking coffee has the ability against SARS-CoV-2 in the cohort study.

**Additional file 2: Table S1.** Vaccination status of each human subject.

## Data Availability

All data generated or analysed during this study are included in this published article and its supplementary information files.
