# Supplementary material for: Coffee as a dietary strategy to prevent SARS-CoV-2 infection
Source: Cell Biosci. 2023 Nov 14;13:210. doi: 10.1186/s13578-023-01154-9 (PMC10644613; doi:10.1186/s13578-023-01154-9)
Supplement: Supplementary file 1 — Additional file 1: Figure S1. Identification of cell viability. Figure S2. Evaluation of the content of compounds in coffee and the inhibiting efficacy against SARS-CoV-2 entry. Figure S3. Ligplot + shows the interactions between Caffeine, CGA, isoCGA-A, isoCGA-B and isoCGA-C with Omicron SP-ACE2 complex amino acids. Hydrogen bonds are shown as green dashed lines. Hydrophobic contacts are represented as spline curves. Figure S4. Ligplot + shows the interactions between Caffeine, CGA, isoCGA-A, isoCGA-B and isoCGA-C with TMPRSS2 amino acids. Hydrogen bonds are shown as green dashed lines. Hydrophobic contacts are represented as spline curves. Figure S5. Drinking coffee has the ability against SARS-CoV-2 in the cohort study. [file 13578_2023_1154_MOESM1_ESM.pdf]

S1 Fig

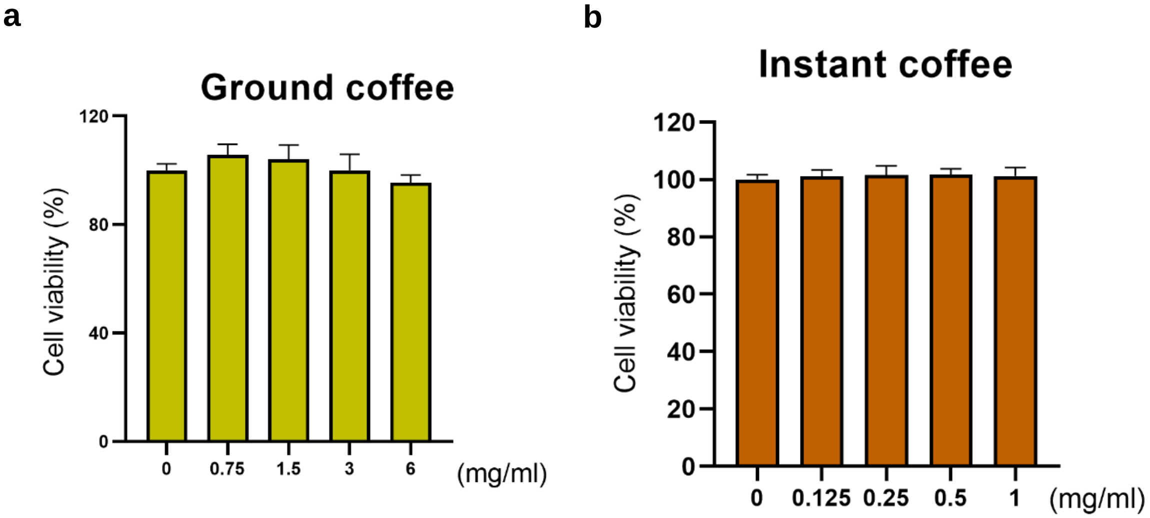

S1 Fig. ***Identification of cell viability.***
The cell viability was tested to verify the safety of ground coffee (a) and instant coffee (b).

S2 Fig

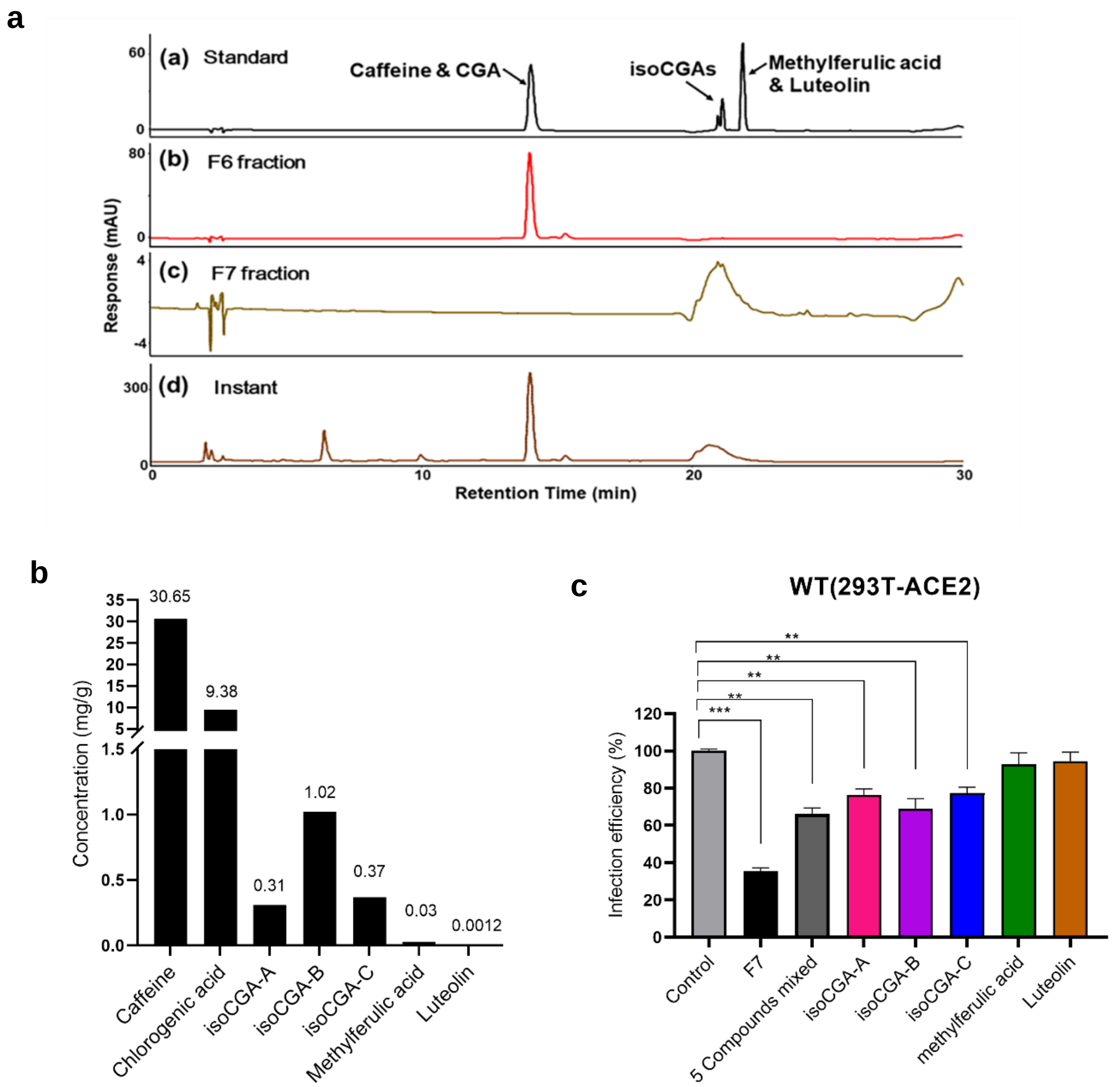

S2 Fig. ***Evaluation of the content of compounds in coffee and the inhibiting efficacy against SARS-CoV-2 entry.***

(a) Compared with the standard of pure compounds, caffeine and chlorogenic acid (CGA) exist in fraction 6, as well as isochlorogenic acid A (isoCGA-A), isochlorogenic acid B (isoCGA-B), isochlorogenic acid C (isoCGA-C), methylferulic acid,and luteolin exist in fraction 7. (b) Analysis the content of compounds in instant coffee by UHPLC-HRMS. (c) According to the content of compounds in coffee, comparing the effect of caffeoylquinic acid derivatives alone, mixed, and fraction 7 on inhibiting SARS-CoV-2 entry by VPP assay.

S3 Fig

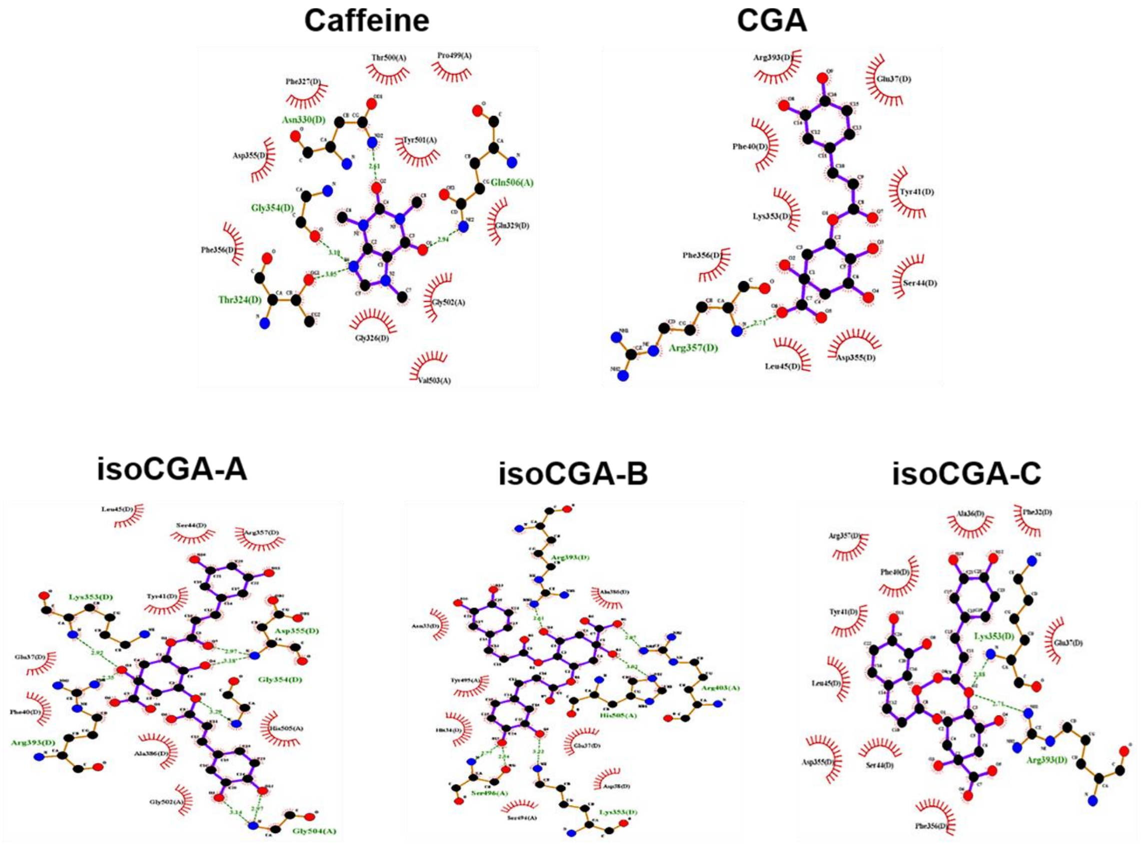

S3 Fig. Ligplot+ shows the interactions between Caffeine, CGA, isoCGA-A, isoCGA-B and isoCGA-C with Omicron SP-ACE2 complex amino acids. Hydrogen bonds are shown as green dashed lines. Hydrophobic contacts are represented as spline curves.

S4 Fig

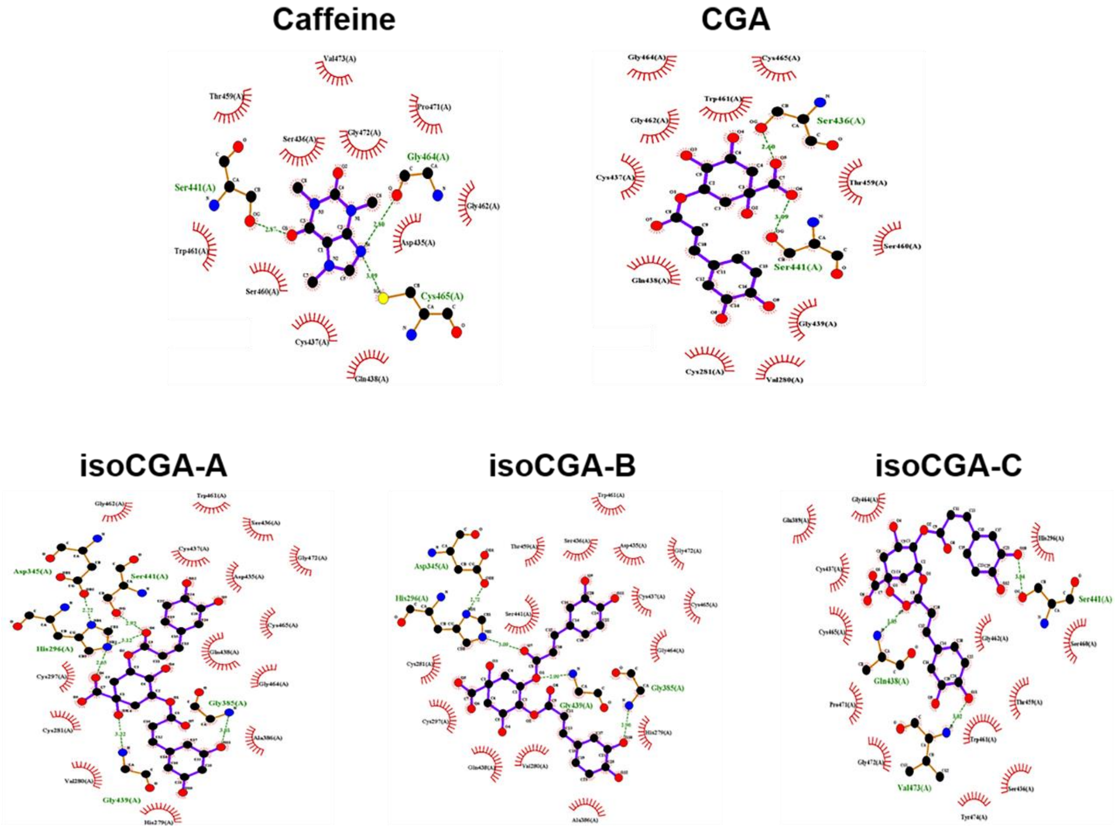

S4 Fig. Ligplot+ shows the interactions between Caffeine, CGA, isoCGA-A, isoCGA-B and isoCGA-C with TMPRSS2 amino acids. Hydrogen bonds are shown as green dashed lines. Hydrophobic contacts are represented as spline curves.

S5 Fig

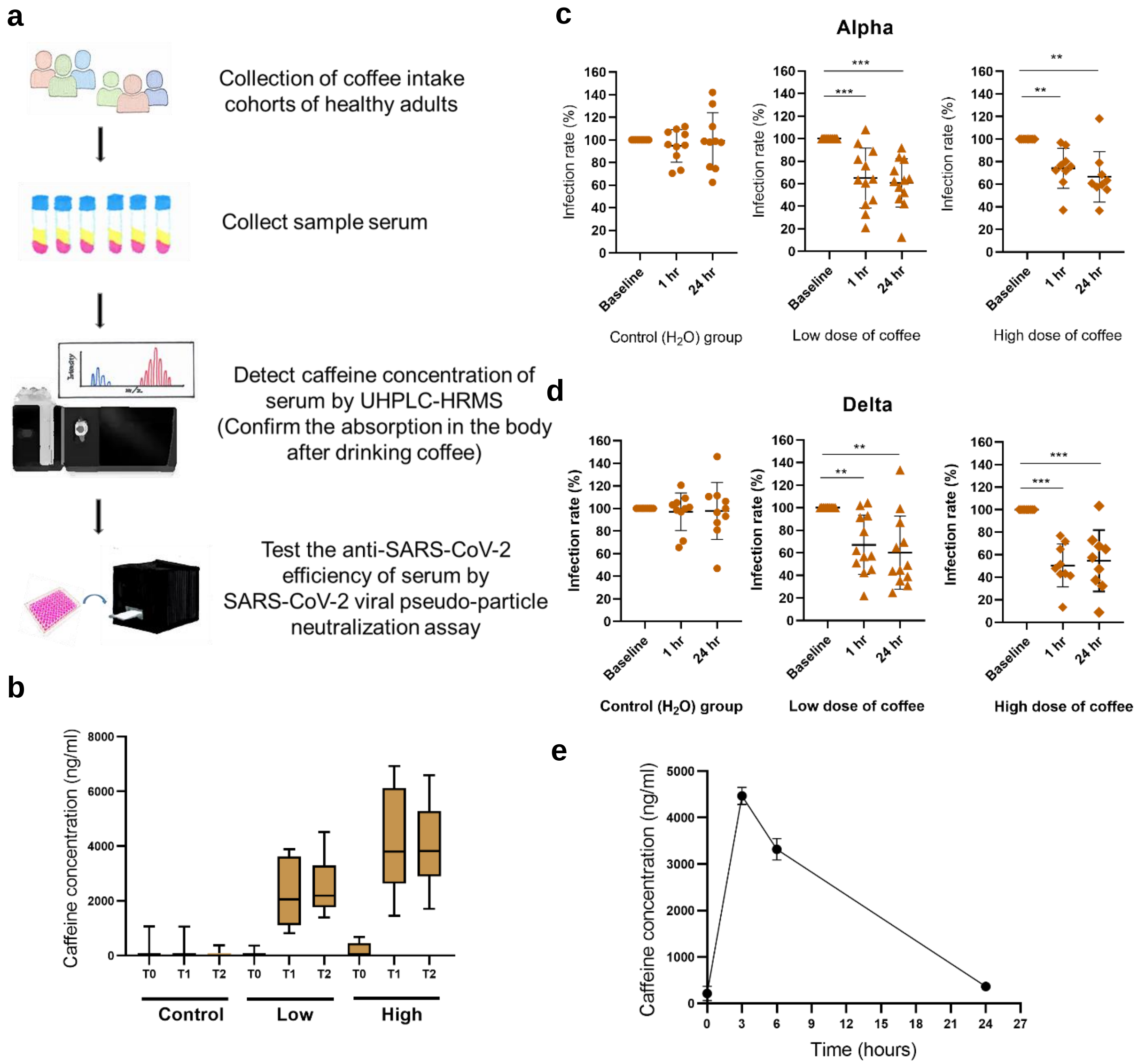

S5 Fig. *Drinking coffee has the ability against SARS-CoV-2 in the cohort study.*

(a)Experimental flow chart of the cohort study. (b) Validation of caffeine metabolic concentrations in the serum of coffee-drinking groups by HPLC analysis. (c) Examination of the serum in drinking coffee with different concentrations against the Alpha variant by Vpp. (d) The efficacy of the serum in drinking coffee with different concentrations against the Delta variant was tested by Vpp. (e) Time course of caffeine metabolism in serum after drinking coffee by HPLC. Statistical significance was concerned as *, $P < 0.05$, **, $P < 0.01$ or ***, $P < 0.001$.
