# Supplementary material for: Coffee as a dietary strategy to prevent SARS-CoV-2 infection
Source: Cell Biosci. 2023 Nov 14;13:210. doi: 10.1186/s13578-023-01154-9 (PMC10644613; doi:10.1186/s13578-023-01154-9)
Supplement: Supplementary file 2 — Additional file 2: Table S1. Vaccination status of each human subject. [file 13578_2023_1154_MOESM2_ESM.docx]

**S1 Table Vaccination status of each human subject.**

|  | **Types of COVID-19 Vaccines** | | | |
| --- | --- | --- | --- | --- |
| **Human subject** | **Dose 1** | **Dose 2** | **Dose 3** | **Dose 4** |
| Control group- No. |  | | | |
| 1 | AZ | BNT | M |  |
| 2 | M | M | BNT |  |
| 3 | M | M | M |  |
| 4 | AZ | AZ | M |  |
| 5 | AZ | M | M |  |
| 6 | BNT | BNT | M |  |
| 7 | M | M | M |  |
| 8 | AZ | AZ | M |  |
| 9 | M | M | BNT |  |
| 10 | M | M | BNT |  |
| Coffee group- No. |  | | | |
| Low dose |  | | | |
| 1 | BNT | BNT | BNT |  |
| 2 | AZ | AZ | M |  |
| 3 | AZ | AZ | BNT |  |
| 4 | AZ | AZ |  |  |
| 5 | AZ | AZ | M | M |
| 6 | M | M | BNT |  |
| 7 | AZ | AZ | M |  |
| 8 | AZ | AZ | M |  |
| 9 | AZ | AZ | M |  |
| 10 | Sinovac | Sinovac | M |  |
| 11 | AZ | AZ | M |  |
| 12 | AZ | AZ | M |  |
| High dose |  | | | |
| 1 | AZ | M | BNT |  |
| 2 | AZ | AZ | M |  |
| 3 | AZ | AZ | M |  |
| 4 | M | M | BNT |  |
| 5 | M | M | M |  |
| 6 | AZ | AZ | M |  |
| 7 | M | M | M |  |
| 8 | M | M | M |  |
| 9 | AZ | AZ | BNT |  |
| Time course |  | | | |
| 1 | AZ | AZ | M |  |
| 2 | M | M | M |  |
| 3 | M | M | M |  |
| 4 | BNT | M |  |  |
| 5 | AZ | AZ | M |  |
| 6 | BNT | BNT |  |  |
| 7 | AZ | AZ | AZ |  |
| 8 | AZ | AZ | MVC |  |
| 9 | MVC | MVC | MVC |  |
| 10 | MVC | MVC | BNT |  |
| 11 | AZ | AZ | MVC |  |
| 12 | AZ | AZ | BNT |  |
| Decaf coffee group-No. |  | | | |
| Low dose |  | | | |
| 1 | AZ | AZ | M |  |
| 2 | BNT | BNT | BNT |  |
| 3 | AZ | AZ | M |  |
| 4 | BNT | BNT | MVC |  |
| 5 | BNT | BNT | M |  |
| 6 | AZ | M | M |  |
| 7 | AZ | AZ | AZ |  |
| 8 | AZ | AZ | M |  |
| 9 | AZ | AZ | M |  |
| High dose |  | | | |
| 1 | AZ | AZ | M |  |
| 2 | AZ | AZ | M |  |
| 3 | MVC | MVC | M |  |
| 4 | AZ | AZ | M |  |
| 5 | MVC | MVC | BNT |  |
| 6 | BNT | BNT | BNT |  |
| 7 | AZ | M | M |  |
| 8 | Novavax |  |  |  |
| 9 | AZ | BNT | BNT |  |
| 10 | AZ | AZ | BNT |  |
| 11 | AZ | AZ | M |  |
| 12 | BNT | BNT | M |  |

AZ, AstraZeneca COVID‑19 vaccine；BNT, Pfizer BioNTech (BNT162b2) COVID-19 vaccine；M, Moderna COVID‑19 vaccine；Sinovac, Sinovac-CoronaVac COVID-19 vaccine；MVC, MVC COVID-19 Vaccine；Novavax, Novavax COVID-19 vaccine.
